# Rapid and visual identification of HIV-1 using reverse transcription loop-mediated isothermal amplification integrated with a gold nanoparticle-based lateral flow assay platform

**DOI:** 10.3389/fmicb.2023.1230533

**Published:** 2023-07-12

**Authors:** Xu Chen, Cheng Du, Qiang Zhao, Qi Zhao, Yonghu Wan, Jun He, Wei Yuan

**Affiliations:** ^1^The Second Clinical College, Guizhou University of Traditional Chinese Medicine, Guiyang, Guizhou, China; ^2^Clinical Medical Laboratory of the Second Affiliated Hospital, Guizhou University of Traditional Chinese Medicine, Guiyang, Guizhou, China; ^3^Department of Anesthesiology, The Second Affiliated Hospital, Guizhou University of Traditional Chinese Medicine, Guiyang, Guizhou, China; ^4^Clinical Laboratory, Guizhou Provincial Center for Clinical Laboratory, Guiyang, Guizhou, China; ^5^Gastroenterology of the Second Affiliated Hospital, Guizhou University of Traditional Chinese Medicine, Guiyang, Guizhou, China; ^6^Experiment Center, Guizhou Provincial Centre for Disease Control and Prevention, Guiyang, Guizhou, China; ^7^Department of Quality Control, Guizhou Provincial Center for Clinical Laboratory, Guiyang, Guizhou, China

**Keywords:** human immunodeficiency virus type one, loop-mediated isothermal amplification, gold nanoparticle-based lateral flow assay, point-of-care platform, limit of detection

## Abstract

Human immunodeficiency virus type one (HIV-1) infection remains a major public health problem worldwide. Early diagnosis of HIV-1 is crucial to treat and control this infection effectively. Here, for the first time, we reported a novel molecular diagnostic assay called reverse transcription loop-mediated isothermal amplification combined with a visual gold nanoparticle-based lateral flow assay (RT-LAMP-AuNPs-LFA), which we devised for rapid, specific, sensitive, and visual identification of HIV-1. The unique LAMP primers were successfully designed based on the *pol* gene from the major HIV-1 genotypes CRF01_AE, CRF07_BC, CRF08_BC, and subtype B, which are prevalent in China. The optimal HIV-1-RT-LAMP-AuNPs-LFA reaction conditions were determined to be 68°C for 35 min. The detection procedure, including crude genomic RNA isolation (approximately 5 min), RT-LAMP amplification (35 min), and visual result readout (<2 min), can be completed within 45 min. Our assay has a detection limit of 20 copies per test, and we did not observe any cross-reactivity with any other pathogen in our testing. Hence, our preliminary results indicated that the HIV-1-RT-LAMP-AuNPs-LFA assay can potentially serve as a useful point-of-care diagnostic tool for HIV-1 detection in a clinical setting.

## Introduction

Human immunodeficiency virus type one (HIV-1) is an important agent that is responsible for acquired immunodeficiency syndrome (AIDS): approximately 38 million people globally live with this virus, and nearly 650,000 people died from AIDS-related illnesses in 2021, according to the Joint United Nations Programme on HIV/AIDS (UNAIDS) (Bernstein and Wegman, 2018; Eisinger and Fauci, 2018; UNAIDS, 2022). HIV-1 infection remains a global public health concern, the UNAIDS initiated the global project termed “95-95-95” to end the global HIV/AIDS epidemic by 2030, stressing the importance of diagnostic tests and aiming for 95% of people living with HIV-1 to know their status, 95% of people with diagnosed HIV-1 infection to receive sustained treatment, and 95% of people on treatment to achieve viral load suppression (Rojas-Celis et al., 2019; Kin-On et al., 2022). Developing an advanced testing system is crucial to meet these goals and timely control the transmission of the disease.

Diagnosis of HIV-1 agent is critical for both the prevention of its transmission and the improvement of antiretroviral therapy efficacy. However, traditional immunoassays may not be suit for detecting acute HIV-1 infection due to having a long window period (3–6 weeks) (Curtis et al., 2012). During this stage, HIV-1-specific antibodies are not yet generated in patients (Feinberg and Keeshin, 2022). Moreover, sero-conversion may be much later than 3–6 week, even when viral loads may have dropped. Real-time polymerase chain reaction (RT–PCR) has been regarded as a major breakthrough and has been most widely used for the early detection of HIV-1 infection (Zhao et al., 2021). However, RT–PCR limits the application of point-of-care (POC) techniques because it requires a relatively sophisticated thermocycler, trained technical personnel, and extended reaction times (approximately 2.5 h) (Li et al., 2021; Zhao et al., 2021). In addition, these tests need to be carried out in specialized and often centralized laboratories. Hence, devising an easy-to-operate, cost-effective, rapid, sensitive, and specific POC assay system is necessary for the prevention and follow-up treatment of HIV-1 infection.

Loop-mediated isothermal amplification (LAMP) is a promising nucleic acid isothermal amplification method that has enormous potential to transform POC molecular diagnostics due to its easy operation, high specificity, sensitivity, and lack of specialized equipment (i.e., PCR machines) (Notomi et al., 2000; Park, 2022; Shirshikov and Bespyatykh, 2022). In principle, LAMP requires a *Bst* DNA polymerase with strand displacement activity and a set of four to six specific primers that recognize different fragments in the target sequence (Notomi et al., 2000). In particular, the amount of amplicon generated from LAMP amplification is usually 100-fold greater than that generated from traditional PCR-based reactions (Avendaño and Patarroyo, 2020; Chaouch, 2021). The LAMP assay has previously been used for the detection of pathogens such as human influenza virus, severe acute respiratory syndrome coronavirus 2, and Zika virus (Ahn et al., 2019; Silva et al., 2019; Li et al., 2022). Moreover, LAMP has already been applied to detect HIV-1. Curtis et al. (2008) combined reverse-transcription LAMP with agarose gel electrophoresis for the identification of HIV-1. Curtis et al. (2008), Li et al. (2022), and Zhang et al. (2022) combined LAMP with a fluorescence reader for the detection of HIV-1. However, these assays require specific facilities for the analysis of LAMP products.

**FIGURE 1:**
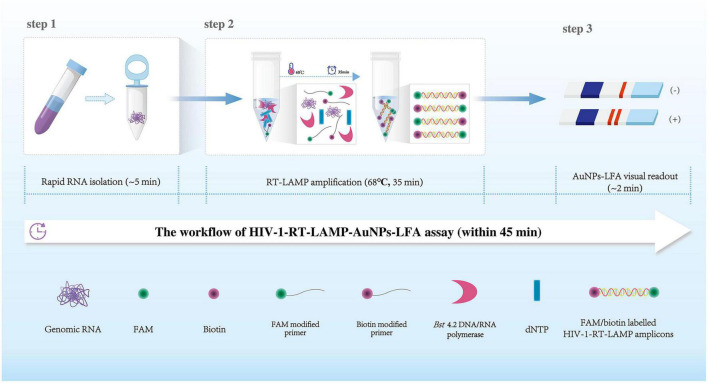
Human immunodeficiency virus type one (HIV-1)-RT-LAMP-AuNPs-LFA assay workflow. The HIV-1-RT-LAMP-AuNPs-LFA assay’s workflow includes genomic RNA isolation (within 5 min), HIV-1-RT-LAMP amplification (35 min), and AuNPs-LFA visual interpretation (<2 min) and can be completed within 45 min. HIV-1, human immunodeficiency virus type one; RT-LAMP, reverse transcription loop-mediated isothermal amplification; AuNPs-LFA, gold nanoparticles-based lateral flow assay.

**FIGURE 2:**
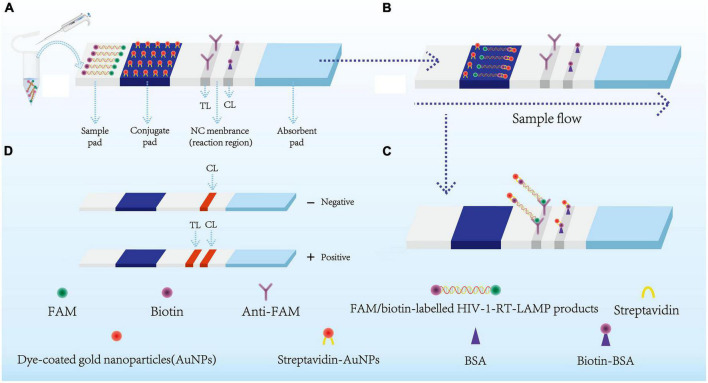
Schematic diagram showing AuNPs-LFA principles for visual HIV-1-RT-LAMP products interpretation. **(A)** HIV-1-RT-LAMP amplification products (2 μl) and running buffer (100 μl) were added simultaneously on the sample pad. **(B)** The FAM/biotin-labeled HIV-1-LAMP products flowing to the conjugate pad through capillary forces. Meanwhile, the streptavidin-AuNPs were hydrated and integrated with HIV-1-RT-LAMP products. **(C)** FAM/biotin-labeled HIV-1-RT-LAMP products were captured with anti-FAM at TL, and streptavidin-AuNPs were captured through biotin-BSA at CL. **(D)** Interpretation of the HIV-1-RT-LAMP-AuNPs-LFA assay. HIV-1 positive results were indicated by CL and TL bands on the AuNPs-LFA, Negative results were indicated when only the CL band appears on the AuNPs-LFA. HIV-1, human immunodeficiency virus type one; RT-LAMP, reverse transcription loop-mediated isothermal amplification; streptavidin-AuNPs, crimson red dye streptavidin-coated gold nanoparticles. AuNPs-LFA, gold nanoparticles-based lateral flow assay. CL, control line; TL, test line.

Gold nanoparticle-based lateral flow assay (AuNPs-LFA) has shown great potential as an ideal POC diagnostic platform due to their superior portability, usability by non-technical personnel, visual interpretation by the naked eye, and rapid detection (Campuzano et al., 2019; Gumus et al., 2023). AuNPs-LFA strips have been widely used to identify various analytes, such as nucleic acids, proteins, and infectious pathogens (Quesada-González and Merkoçi, 2015; Ince and Sezgintürk, 2022; Sohrabi et al., 2022). In the current study, the RT-LAMP reaction was combined with a AuNPs-based LFB detector (RT-LAMP-AuNPs-LFA) to develop an advanced assay for specific, sensitive, rapid, visual, and cost-saving identification of HIV-1 by targeting its *pol* gene (Zhao et al., 2021), which showed has no homology to other pathogen genomes in BLAST searches of the GenBank database. The RT-LAMP-AuNPs-LFA principle and workflow are shown in Figures 1, 2, respectively. The feasibility of our assay was verified through clinical serum samples from suspected HIV-1-infected patients.

## Materials and methods

### Reagents

AuNPs-LFA-related materials, including crimson red dye streptavidin-coated AuNPs (SA-AuNPs; 40 ± 5 nm, 10 mg/mL), were obtained from Bangs Laboratories Inc. (Fishers, IN, USA), rabbit anti-fluorescein antibody (anti-FAM; 0.2 mg/mL) and biotinylated bovine serum albumin (biotin-BSA; 4 mg/mL) were obtained from Abcam Co., Ltd. (Shanghai, China), and nitrocellulose membrane (NC) was obtained from Merck Millipore Co., Ltd. (Darmstadt, Germany). Four components of the AuNPs-LFA, including the sample, conjugate, absorption pads, and nitrocellulose membranes, were manufactured and laminated on plastic adhesive backing by HuiDeXin Biotech. Co., Ltd. (Tianjin, China) according to our design scheme (Figure 2). Nucleic acid releasing agents were obtained from GenDx Biotech Co., Ltd. (Suzhou, China). LAMP amplification kits and colorimetric indicator (Leuco-hydroxynaphthol blue, L-HNB) were obtained from HaiGene Biotech Co., Ltd. (Harbin, China). Commercial RT–qPCR diagnostic kits for HIV-1 were obtained from DaAn Gene Co., Ltd. (Guangzhou, China).

### Clinical specimens and target gene preparation

In this study, 65 clinical serum specimens were collected from suspected HIV-infected patients from Guizhou Provincial Center for Clinical Laboratory between January 2023 and April 2023. Genomic RNA was isolated using Nucleic Acid Releasing Agent (GenDx Biotech Co., Ltd.) in accordance with the manufacturer’s instructions.

The full-length *pol* gene sequences of the representative genotypes of all known HIV-1 prevalent genotypes, including CRF01_AE, CRF07_BC, CRF08_BC, and subtype B, in China were downloaded from the GenBank database (genotype CRF01_AE: GenBank Accession No. U54771.1; genotype CRF07_BC: GenBank Accession No. U54771.1; genotype CRF08_BC: GenBank Accession No. AY008715.1; genotype subtype B: GenBank Accession No. GU647198.1) (He et al., 2012; Zhao et al., 2018). The four genomic sequences were synthesized and cloned into the pUC57 vector by Tsingke Biotech (Beijing, China). The initial concentration of each plasmid was 1 × 10^8^ copies per milliliter, and the HIV-1 genotype CRF01_AE plasmid was used as a positive control.

### AuNPs-based LFB construction

A schematic of the AuNPs-based LFA used in this study is shown in Figure 2. Briefly, the AuNPs-LFA (60 mm × 4 mm) is composed of four sections, including the sample pad, conjugate pad, detection region (nitrocellulose membrane), and absorption pad. Crimson red dye streptavidin-coated gold nanoparticles (SA-AuNPs) were deposited on the conjugate pad. Rabbit anti-FAM (0.2 mg/mL) and biotin-BSA (4 mg/mL) were fixed onto the nitrocellulose membrane of the test line (TL) and control line (CL), respectively, and the two lines were separated by 5 mm. In the end, the four separate sections were combined together on a plastic card through adhesive backing. The AuNPs-based LFA was kept dry and at room temperature until use.

### LAMP primer design

The HIV-1 *pol* gene was selected as the amplification target for the RT-LAMP-AuNPs-LFA assay. The *pol* gene sequences from representative genotypes of all known HIV-1 prevalent genotypes (CRF01_AE, CRF07_BC, CRF08_BC, and subtype B) in China were aligned with DNASTAR software (DNASTAR Inc., Madison, WI, USA) (Supplementary Figure 1). The conserved sequences were used for HIV-1 RT-LAMP primers design through Primer Explorer v.5^1^ and Primer Premier v.5.0 software. The specificity of the primer set was verified with the BLAST analysis tool. The primer sequences and alterations designed in this study are summarized in Table 1, and the primer locations are shown in Supplementary Figure 1. All primers were synthesized and purified via high-performance liquid chromatography at TsingKe Biotech Co., Ltd. (Beijing, China).

**TABLE 1:** The HIV-1-RT-LAMP-AuNPs-LFA primers used in this study.

Primer name	Sequence and modifications	Length	Gene
F3	5′-ATGGCAGTATTCAT(T/C)CACAAT-3′	21 nt	*pol*
B3	5′-CTACTGCCCCTTCACCT-3′	17 nt	
FIP	5′-GTATGTCTGTTGCTATTAT(G/A)TCTA(T/C)-TAAAAGAAAAGGGGGGATTGG-3′	46 mer	
FIP*	5′-FAM-GTATGTCTGTTGCTATTAT(G/A)TCTA(T/C)-TAAAAGAAAAGGGGGGATTGG-3′	46 mer	
BIP	5′-TCAAAATTTTCGGGTTTATTACAG(G/A)- AG(T/G)AG(T/C)TT(T/)GCTGGTCCTT-3′	44 mer	
LF	5′-TCTTTCCCCTGCACTGTAC-3′	19 nt	
LF*	5′-Biotin-TCTTTCCCCTGCACTGTAC-3′	19 nt	
LB	5′-ACAGCAGAGA(C/T)CC(A/C)(A/C)(T/G)TTG-3′	19 nt	

*pol*-FIP*, 5′-labeled with FAM, *pol*-LF*, 5′-labeled with biotin, when used for the AuNPs-LFA assay. FAM, 6-carboxy-fluorescein; nt, nucleotide; mer, monomeric unit.

### Standard HIV-1-RT-LAMP-AuNPs-LFA reaction

The RT-LAMP reaction for HIV-1 was performed in 25 μl volumes containing 2.5 μl of 10 × *Bst* 4.2 Buffer (Mg^2+^ free); 1.5 μl of 100 mM Mg^2+^; 3 μl of dNTP Mixture (10 mM each); 0.1 μM of F3 and B3 primers; 0.4 μM of FIP or FIP* (for AuNPs-LFA only) and BIP primers; 0.2 μM of LF or LF* (for AuNPs-LFA only) and LB; 1 μl of *Bst* 4.2 DNA/RNA polymerase (8 U); 1.5 μl of L-HNB (for colorimetry only); and 1 μl of plasmid DNA (5 μl of clinical sample template); with double-distilled water added to bring the volume to 25 μl. The reactions were carried out at a constant temperature (reaction conditions were optimized as outlined below).

The LAMP products were tracked through agarose gel electrophoresis, real-time turbidity, colorimetry (L-HNB), and AuNPs-LFA. Briefly, for positive results, the agarose gel presented ladder-like bands, while there have no bands in negative outcomes. A turbidity value of >0.1 indicated a successful outcome. A reaction mixture that turned from deep violet to light green indicated successful L-HNB visual detection, and the mixture that remained deep violet throughout the reaction indicated a negative result. For AuNP-LFB detection, the simultaneous appearance of CL and TL on the AuNPs-LFA indicated a positive HIV-1-LAMP result or a negative outcome with only CL present on the AuNPs-LFA.

### HIV-1-RT-LAMP-AuNPs-LFA assay condition optimization

RT-LAMP reaction temperatures from 63 to 70°C (in 1°C increments) were tested to confirm the optimal temperature under the standard HIV-1-RT-LAMP reaction system, and the amplification results were assessed using real-time turbidity. Then, the optimal reaction time was determined by incubating the HIV-1-RT-LAMP reactions at 15 to 45 min (in 10 min increments) and detecting their amplification results through AuNPs-LFA and L-HNB visual reagent. Each test was performed in triplicate.

### HIV-1-RT-LAMP-AuNPs-LFA assay sensitivity

The *pol* plasmids were produced and serially diluted 10-fold (2.0 × 10^4^ to 2.0 × 10^–1^ copies) to confirm the limit of detection (LoD) of the HIV-1-RT-LAMP-AuNPs-LFA assay. The HIV-1-RT-LAMP reactions were performed under optimum conditions, and the amplification results were assayed using AuNPs-LFA and L-HNB. Each test was repeated three times.

### HIV-1-RT-LAMP-AuNPs-LFA assay specificity

Four synthetic *pol*-plasmid DNA templates (HIV-1 genotypes CRF01_AE, CRF07_BC, CRF08_BC, and subtype B) and other bacterial, viral, and fungal nucleic acid templates at ≥ 1.0 × 10^4^ copies were used to evaluate the HIV-1-RT-LAMP-AuNPs-LFA assay’s specificity (Table 2). Distilled water (DW) served as a negative control, and the amplification results were tested using AuNPs-LFA. Each assay was performed in triplicate.

**TABLE 2:** Pathogens used in this study.

No.	Pathogen	Source of pathogens^a^	No. of strains	HIV-1-LAMP- AuNPs LFB result^b^
1	HIV-1 CRF01_AE *pol*-plasmid	Constructed by Tsingke Biotech (Beijing, China)	1	P
2	HIV-1 CRF07_BC *pol*-plasmid	Constructed by Tsingke Biotech (Beijing, China)	1	P
3	HIV-1 CRF08_BC *pol*-plasmid	Constructed by Tsingke Biotech (Beijing, China)	1	P
4	HIV-1 subtype B *pol*-plasmid	Constructed by Tsingke Biotech (Beijing, China)	1	P
5	HIV-1 clinical samples	GZCCL	7	P
6	Hepatitis B virus	2nd GZUTCM	1	N
7	Hepatitis C virus	2nd GZUTCM	1	N
8	Influenza A virus	GZCDC	1	N
9	Influenza B virus	GZCDC	1	N
10	Coxsackie virus CAV16	GZCDC	1	N
11	Human enterovirus EV71	GZCDC	1	N
12	Human papillomavirus	2nd GZUTCM	1	N
13	Epstein-Barr virus	2nd GZUTCM	1	N
14	*Mycobacterium tuberculosis*	GZCDC	1	N
15	*Streptococcus pneumoniae*	2nd GZUTCM	1	N
16	*Pseudomonas aeruginosa*	ATCC27853	1	N
17	*Chlamydia trachomatis*	2nd GZUTCM	1	N
18	*Neisseria gonorrhoeae*	2nd GZUTCM	1	N
19	*Ureaplasma urealyticum*	2nd GZUTCM	1	N
20	*Staphylococcus aureus*	ATCC25923	1	N
21	*Escherichia coli*	ATCC25922	1	N
22	*Enterococcus faecalis*	2nd GZUTCM	1	N
23	*Cryptococcus neoformans*	ATCC 13690	1	N
24	*Shigella boydii*	2nd GZUTCM	1	N
25	*Streptococcus glabra*	2nd GZUTCM	1	N
26	*Haemophilus influenzae*	ATCC49247	1	N
27	*Streptococcus albus*	2nd GZUTCM	1	N
28	*Stenotrophomonas maltophilia*	2nd GZUTCM	1	N
29	*Salmonella typhimurium*	2nd GZUTCM	1	N
30	*Salmonella enteritidis*	2nd GZUTCM	1	N
31	*Acinetobacter lwoffii*	2nd GZUTCM	1	N
32	*Enterobacter aerogenes*	2nd GZUTCM	1	N
33	*Brucella*	GZCDC	1	N

^a^ATCC, American Type Culture Collection; 2nd GZUTCM, the Second Affiliated Hospital, Guizhou University of Traditional Chinese Medicine; GZCCL, Guizhou Provincial Center for Clinical Laboratory; GZCDC, Guizhou Provincial Center for Disease Control and Prevention.

^b^P, positive; N, negative.

### Feasibility of HIV-1-RT-LAMP-AuNPs-LFA for clinical samples

Serum specimens were collected from 65 suspected HIV-1-infected patients at Guizhou Provincial Center for Clinical Laboratory. Genomic RNA templates were obtained rapidly with Nucleic Acid Releasing Agents (GenDx Biotech Co., Ltd; Suzhou, China; Cat No. NR202) according to the manufacturer’s guidelines. Briefly, 150 μl of serum specimens were added into 1.5 ml EP tube, then the nucleic acid releasing agent RNA LB1 (120 μl) and RNA BB2 (500 μl) were added into the specimens for 20 s to release nucleic acid, and then the nucleic acids were collected through centrifuge absorption column. After washing steps, the nucleic acids were eluted in to 30 μl nuclease free H_2_O. the genomic RNA was stored at −80°C before use. The Human Ethics Committee of Guizhou Provincial Center for Clinical Laboratory approved the lawful and ethical collection and analyses. All the samples were detected with RT–qPCR and our HIV-1-LAMP-AuNPs-LFA assay. HIV-1 RT–qPCR detection was performed using HIV-1 Nucleic Acid Assay Kits (DaAn Gene Co., Ltd.; Guangzhou, China) on an Applied Biosystems™ 7500 Real-Time PCR System (Life Technologies; Singapore). The limit of detection (LoD) of this assay was 50 IU/ml (approximately 30 copies/ml), and the basis for determining the copy number of the samples according to the corresponding standard curve. The negative and positive accordance rate of this assay was 97.76 and 99.80%, respectively, according to the manufacturer’s guidelines. In addition, the HIV-1-positive samples were amplified with nested PCR by targeting the *pol* gene, and then the nucleotide sequences were aligned with reference sequences in HIV databases^2^ for HIV-1 subtyping (Njouom et al., 2003; Zhao et al., 2018). The HIV-1-RT-LAMP-AuNPs-LFA assay was performed as previously described. The identification investigations were performed at biosafety level 2 based on the WHO Biosafety Manual (3rd Edition). The HIV-1-LAMP-AuNPs-LFA data were compared with RT–qPCR results. The statistical parameters were calculated using online tool from MedCalc^3^ (Jevtuševskaja et al., 2016).

## Results

### Schematic mechanism of the HIV-1-RT-LAMP-AuNPs-LFA assay

A representative schematic and workflow of the HIV-1-RT-LAMP-AuNPs-LFA assay is shown in Figure 1. In brief, genomic RNA templates were rapidly extracted within 5 min (Figure 1, step 1). The RT-LAMP technique was performed to rapidly and specifically amplify the target gene at a constant temperature (68°C) within 35 min.

The RT-LAMP method amplified the target sequence by using only a *Bst* 4.2 DNA/RNA polymerase with strand displacement activity and a set of 6 primers, including two outer primers (F3 and B3), two inner primers (FIP and BIP), and two loop primers (LF and LB). The FIP* and LF* primers were 5′-labeled with fluorescein (FAM) and biotin, respectively. The RT-LAMP products were labeled with FAM and biotin (Figure 1, step 2). Finally, the HIV-1-RT-LAMP products were visually interpreted with an AuNPs-LFA within 2 min (Figure 1, step 3). The whole assay can be completed within 45 min.

The principle of AuNPs-LFA analysis of HIV-1-RT-LAMP amplification products is presented in Figure 2. Briefly, HIV-1-RT-LAMP products (2.0 μl) and running buffer (100 μL; 100 mM phosphate-buffered saline with 1% Tween 20 [pH 7.4]) were dripped concurrently into the sample pad of AuNPs-LFA (Figure 2A). Capillary action carries the RT-LAMP product-containing running buffer move along the AuNPs-LFA platform, then the streptavidin-dye coated gold nanoparticles (streptavidin-AuNPs) were rehydrated and combined with FAM/biotin-labeled HIV-1-RT-LAMP products at the conjugate pad (Figure 2B). In the reaction region, the anti-FAM was anchored at test line (TL) and used to arrest FAM/biotin-labeled HIV-1-RT-LAMP products, the biotin-BSA was fixed at control line (CL) and used to capture streptavidin-AuNPs (Figure 2C). The interpretation of the HIV-1-RT-LAMP-AuNPs-LFA assay is outlined in Figure 2D. In a positive result, both CL and TL appeared simultaneously on the biosensor, and the result was negative when only CL was present on the biosensor.

### HIV-1-RT-LAMP-AuNPs-LFA assay confirmation

**FIGURE 3:**
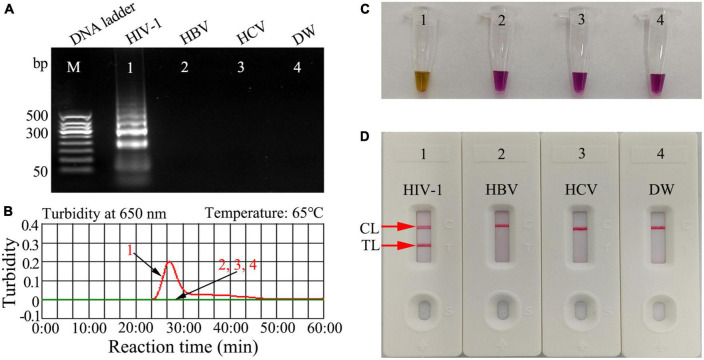
Human immunodeficiency virus type one (HIV-1)-RT-LAMP products verification. The HIV-1-RT-LAMP products were identified simultaneously using **(A)** 2% agarose gel electrophoresis, **(B)** real-time turbidity, **(C)** color change (L-HNB), and **(D)** AuNPs-LFA. Templates of A1/B1/C1/D1 -A4/B4/C4/D4 were the HIV-1-plasmid (positive), HBV (negative), HCV (negative), and DW (blank control), respectively. HIV-1, human immunodeficiency virus type one; HBV, hepatitis B virus; HCV, hepatitis C virus; DW, distilled water; L-HNB, Leuco-hydroxynaphthol blue; CL, control line; TL, test line.

To confirm the feasibility of the HIV-1-RT-LAMP-AuNPs-LFA assay, the HIV-1-RT-LAMP-amplification mixes were incubated at a constant temperature (65°C) for 1 h, and then, the amplification products were analyzed using 2% agarose gel electrophoresis, real-time turbidity, colorimetric indicator (visual detection reagent, L-HNB) and AuNPs-LFA. For agarose gel electrophoresis identification, only the agarose gel of HIV-1-LAMP presented ladder-like bands, and the negative and blank controls have no bands were observed (Figure 3A). The turbidity of HIV-1-LAMP at the positive control was >0.1 and regarded as a positive outcome, while the negative and blank controls were <0.1 and considered negative reactions (Figure 3B). For colorimetric indicator (L-HNB) detection, the positive HIV-1-LAMP reaction changed from deep violet to light green, and the negative and blank controls remained deep violet (Figure 3C). More importantly, two clearly visible crimson-red bands (TL and CL) were observed in the AuNPs-LFA, indicating a positive HIV-1-RT-LAMP reaction, while only the CL was observed in the negative and blank controls (Figure 3D). These data indicated that the LAMP primer set designed for HIV-1 detection was an appropriate candidate for the development of the HIV-1-RT-LAMP-AuNPs-LFA assay.

### HIV-1-RT-LAMP amplification temperature optimization

**FIGURE 4:**
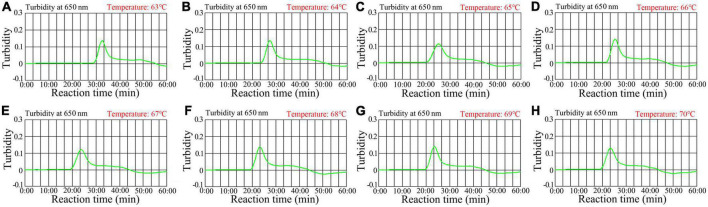
Temperature optimization for the HIV-1-RT-LAMP amplification. RT-LAMP amplifications for HIV-1 were monitored using real-time turbidity, and their corresponding amplicon curves are shown as graphs, a turbidity > 0.1 indicated a positive result. Eight kinetic graphs were obtained at different temperatures (63°C–70°C in 1°C increments) with 2 × 10^3^ target gene copies **(A–H)**. Graphs **(F–H)** (68–70C) showed robust amplification.

Optimizing the reaction temperature is critical for high-efficiency LAMP amplification. In this study, we used a standard *pol*-plasmid copy number (2.0 × 10^3^) to test reaction temperatures from 63 to 70°C with 1°C increments. The HIV-1-RT-LAMP reactions were tracked by means of real-time turbidity measurement, and the kinetic graph was generated from each reaction temperature. The results indicated that robust HIV-1-RT-LAMP amplification was observed at 68 to 70°C (Figure 4). Therefore, 68°C was considered the optimal reaction temperature for our assay.

### HIV-1-RT-LAMP-AuNPs-LFA assay sensitivity

**FIGURE 5:**
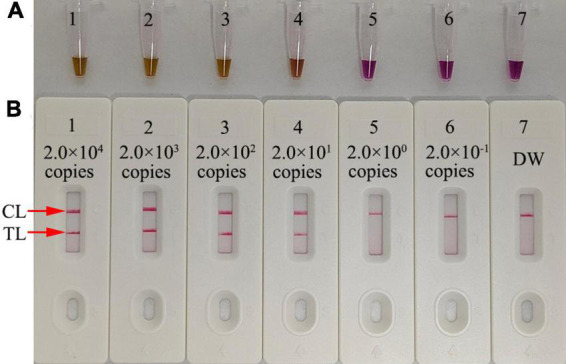
Serial dilutions (2.0 × 10^4^, 2.0 × 10^3^, 2.0 × 10^2^, 2.0 × 10^1^, 2.0 × 10^0^, and 2.0 × 10^−1^ copies) of HIV-1 plasmids were used as templates, and DW was used as the blank control. Results were simultaneously analyzed through visual reagent L-HNB **(A)** and the AuNPs-LFA **(B)**. A sensitivity analysis of the HIV-1-LAMP assay indicated its LoD was 20 copies per test. DW, distilled water; CL, control line; TL, test line.

Serial dilutions of *pol* plasmid with 2.0 × 10^4^ to 2.0 × 10^–1^ copies were used as templates to determine the LoD of our assay. HIV-1-RT-LAMP reactions were carried out at 68°C for 1 h, and the results were monitored using a colorimetric indicator (L-HNB) and AuNPs-LFA. The results obtained via the AuNPs-LFA platform were in accordance with those obtained through the L-HNB method (Figures 5A, B), and the LoD of our assay was 20 copies per test based on HIV-1 DNA.

### HIV-1-RT-LAMP-AuNPs-LFA assay reaction time optimization

**FIGURE 6:**
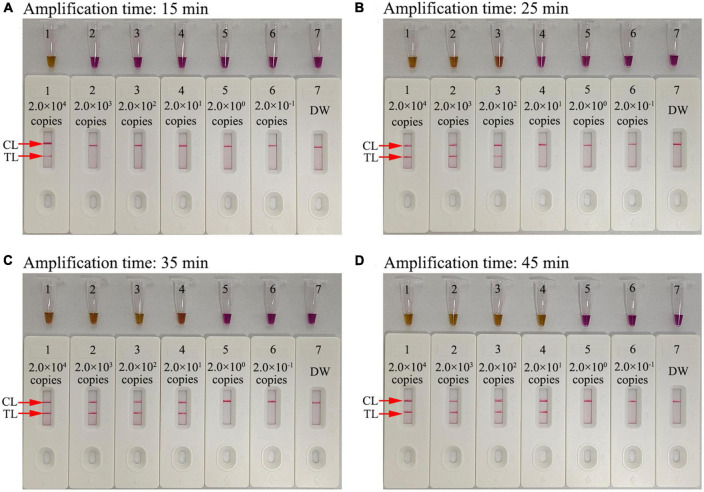
Amplification time optimization for the HIV-1-RT-LAMP-AuNPs-LFA assay. Four RT-LAMP reaction times were evaluated at 68°C: **(A)** 15 min, **(B)** 25 min, **(C)** 35 min, and **(D)** 45 min. Tube/biosensor 1-7 represent nucleic acid template levels 2.0 × 10^4^, 2.0 × 10^3^, 2.0 × 10^2^, 2.0 × 10^1^, 2.0 × 10^0^, and 2.0 × 10^−1^ copies and blank control (DW), respectively. Results were analyzed using visual reagent L-HNB and AuNPs-LFA. The optimal LoD occurred with a 35 min reaction time. DW, distilled water; CL, control line; TL, test line.

The optimal reaction time required for our assay at the HIV-1-RT-LAMP amplification stage was evaluated by testing times from 15 to 45 min in 10 min increments at the optimal amplification temperature (68°C), and the LAMP products were analyzed through a colorimetric indicator (L-HNB) and the AuNPs-LFA platform. The results showed that the LoD of the *pol* plasmid (20 copies) was detected when the reaction time was 35 min (Figure 6). Hence, an HIV-1-RT-LAMP reaction time of 35 min was recommended for our assay. As a result, the whole detection procedure for the HIV-1-RT-LAMP-AuNPs-LFA assay, including rapid template isolation (approximately 5 min), LAMP reaction (35 min), and result visual reporting with AuNPs-LFA (within 2 min), can be completed within 45 min.

### HIV-1-RT-LAMP-AuNPs-LFA assay specificity

**FIGURE 7:**
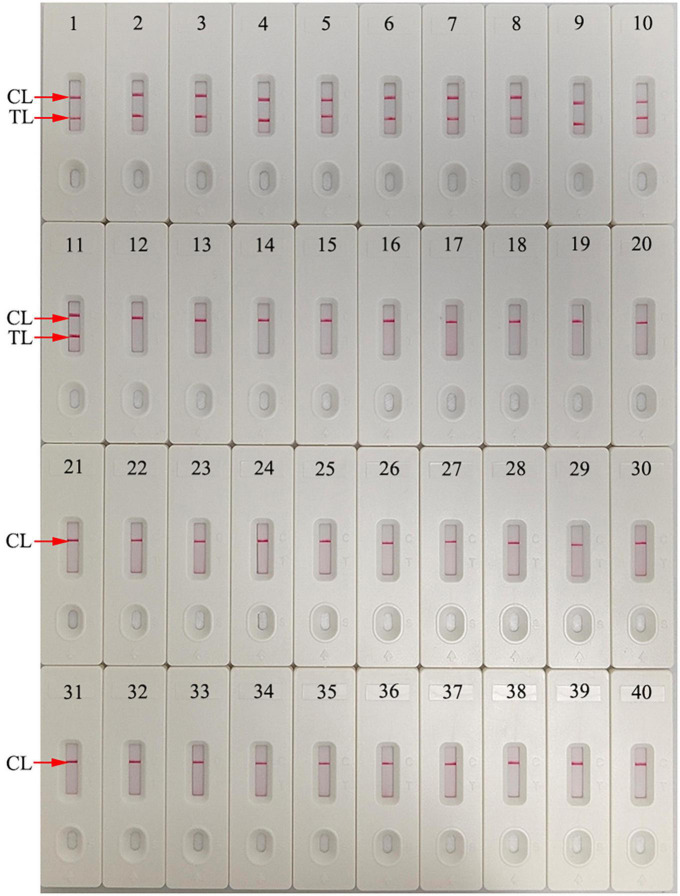
Human immunodeficiency virus type one (HIV-1)-RT-LAMP-AuNPs-LFA assay specificity with different strains. Assay specificity was evaluated using different nucleic acid templates. Amplification products were tested using AuNPs-LFA: 1–4, HIV-1 CRF01_AE, CRF07_BC, CRF08_BC, and subtype B pol plasmids; 5-11, HIV-1 clinical samples; 12, hepatitis B virus; 13, hepatitis C virus; 14, influenza A virus; 15, influenza B virus; 16, Coxsackie virus CAV16; 17, human enterovirus EV71; 18, human papillomavirus; 19, Epstein-Barr virus; 20, *Mycobacterium tuberculosis*; 21, *Streptococcus pneumoniae*; 22, *Pseudomonas aeruginosa*; 23, *Chlamydia trachomatis*; 24, *Neisseria gonorrhoeae*; 25, *Ureaplasma urealyticum*; 26, *Staphylococcus aureus*; 27, *Escherichia coli*; 28, *Enterococcus faecalis*; 29, *Cryptococcus neoformans*; 30, *Shigella boydii*; 31, *Streptococcus glabra*; 32, *Haemophilus influenzae*; 33, *Streptococcus albus*; 34, *Stenotrophomonas maltophilia*; 35, *Salmonella typhimurium*; 36, *Salmonella enteritidis*; 37, *Acinetobacter lwoffii*; 38, *Enterobacter aerogenes*; 39, *Brucella*; 40, distilled water (Blank control). CL, control line; TL, test line.

The specificity of our assay was evaluated using four *pol* plasmids of HIV-1 genotypes (CRF01_AE, CRF07_BC, CRF08_BC, and subtype B) prevalent in China, positive HIV-1 clinical samples (confirmed by RT-qPCR), and 28 other pathogens (Table 2). The HIV-1-RT-LAMP-AuNPs-LFA reactions were performed under optimal conditions, and the results were monitored using the AuNPs-LFA platform. The nucleic acid isolated from HIV-1 samples presented positive results, while other pathogens and blank control groups showed negative results (Table 2 and Figure 7). These data demonstrated that our assay had excellent specificity and no cross-reactivity with non-HIV-1 pathogens.

### Feasibility of HIV-1-RT-LAMP-AuNPs-LFA assay in clinical specimens

To further evaluate the suitability of our assay as a valuable tool for HIV-1 detection, the HIV-1-RT-LAMP-AuNPs-LFA system was tested using 65 suspected HIV-1-infected serum specimens collected from the Guizhou Provincial Center for Clinical Laboratory (Guiyang, China), and the Human Ethics Committee of Guizhou Provincial Center for Clinical Laboratory approved the lawful and ethical collection and analyses. All clinical specimens were tested simultaneously using RT–qPCR and our assay. The results showed that 38 of 65 samples were diagnosed as HIV-1 positive with RT–qPCR (>30 copies). We also analyzed the genotypes of HIV-1-positive samples through nested PCR and sequencing. The results showed that the HIV-1-LAMP-AuNPs-LFA assay was consistent with traditional RT–qPCR testing outcomes (Table 3 and Supplementary Table 1). Comparing with the RT–qPCR technology, the HIV-1-RT-LAMP-AuNPs-LFA sensitivity and specificity was 100% (95% CI: 90.75 to 100.00%) and 100% (95% CI: 87.23 to 100.00%), respectively (Table 3). These data indicated that our assay developed in the current study is a valuable clinical diagnostic tool for HIV-1.

**TABLE 3:** Comparing HIV-1 levels in clinical samples using RT-qPCR and our HIV-1-RT-LAMP-AuNPs-LFA methods.

HIV-1-LAMP-AuNPs-LFA	HIV-1 RT-qRCR (reference method)			Sensitivity		Specificity	
	Positive	Negative	Total	Value	95% CI	Value	95% CI
Positive	38 (CRF01_AE, 16; CRF07_BC, 14; CRF08_BC, 4; Subtype B, 4)	0	38	100%	90.75–100.00%	100%	87.23–100.00%
Negative	0	27	27				
Total	38	27	65				

## Discussion

In this study, we successfully developed and verified a novel HIV-1-RT-LAMP-AuNPs-LFA POC testing system to identify HIV-1, which dexterously integrated HIV-1 specific and rapid RT-LAMP amplification with a visual and sensitive AuNPs-LFA readout platform. The feasibility of our novel assay was confirmed through clinical sera from individuals with suspected HIV-1 infection, and its results were compared with a valuable commercial RT–qPCR assay.

Human immunodeficiency virus type one is one of the major human viruses that can affect the ability of the immune system to defend against life-threatening infections (Inamdar et al., 2019; Pedro et al., 2019; Gandhi et al., 2023). The clinical symptoms are often difficult to distinguish from those associated with the common fevers, muscle pains, and rash at the early stage of HIV-1 infection (Bernstein and Wegman, 2018; Rhee et al., 2022). A rapid, sensitive, specific, easy-to-operate, and cost-saving diagnosis system that can provide early viral detection is critical for prescribing more effective antiretroviral treatments (ARTs) and preventing HIV-1 transmission. Here, Our HIV-1-RT-LAMP-AuNPs-LFA assay only requires basic facilities, such as a heating block, metal bath, water bath, or even a thermos cup, that can hold the reaction temperature at 68°C for the HIV-1-RT-LAMP preamplification step. In our detection system, crude nucleic acid is sufficient for LAMP amplification because the *Bst* 4.2 DNA/RNA polymerase is used for LAMP amplification, which has fewer inhibitors than the *Taq* DNA polymerase used in traditional PCR (Somboonna et al., 2018). Hence, our assay is time-saving, and the entire procedure, including crude genomic RNA isolation (∼5 min), RT-LAMP amplification (35 min), and AuNPs-LFA visual result interpretation (<2 min), can be completed within 45 min.

In our study, the target gene HIV-1 *pol* was amplified using the RT-LAMP technique, which can robustly amplify amplicons at a constant temperature and provide 100-fold greater detection capability than traditional PCR (Avendaño and Patarroyo, 2020; Chaouch, 2021). The specific amplicons were generated in the LAMP reaction system through the *Bst* 4.2 DNA/RNA polymerase with six specific primers that span eight unique segments of the target gene. Here, we successfully designed a set of unique primers based on the four main prevalent HIV-1 genotypes (CRF07_BC, CRF01_AE, CRF08_BC and subtype B) in China for specific amplification of the HIV-1 *pol* gene (He et al., 2012; Zhao et al., 2018). The primer set included two outer primers (F3 and B3), two inner primers (FIP and BIP), and two loop primers (LF and LB). The specificity of the HIV-1-RT-LAMP-AuNPs-LFA assay was verified with HIV-1 strain and 28 other pathogens. Our results confirmed that our assay can correctly identify the target pathogen HIV-1 and has no cross-reaction with non-HIV-1 strains (Table 2 and Figure 7). In addition, the LoD of our assay was as low as 20 copies based on HIV-1 DNA (Figure 5). To further verify the feasibility of our assay in clinical practice, 65 genomic RNA specimens were extracted from suspected HIV-1-infected patients and then analyzed simultaneously using RT–qPCR and our assay. The data confirmed that our assay can effectively identify clinical samples. Furthermore, a next trial should test more clinical samples with significantly lower viral loads with our assay to better reflect its clinical applications.

For a visual and convenient readout of the HIV-1-RT-LAMP amplification results, a AuNPs-LFA platform was constructed and applied in our assay. AuNPs-LFA, as a paper-based biosensor, fits the requirements of POC testing owing to its good selectivity, high sensitivity, low limit of detection, quick assay performance, low cost, and low sample volume (Henderson et al., 2018; Bishop et al., 2019; Boehringer and O’Farrell, 2021). More importantly, AuNPs are the most suitable nanomaterial for use as an optical label in biosensors due to their biocompatibility, ease of synthesis, size-tunability, and intense red color, which is easy to detect by the naked eye (Quesada-González and Merkoçi, 2015; Li et al., 2023). The AuNPs-LFA can readout visually the HIV-1-RT-LAMP results for anchoring anti-FAM and BSA-biotin on the NC membrane. If HIV-1-RT-LAMP positive products presented in the sample, they will be integrated with streptavidin-AuNPs at the conjugate pad. And then, passing through the NC membrance, FAM/biotin-labeled HIV-1-RT-LAMP products will be captured at TL. And to the CL, being always captured for evidence that the streptavidin-AuNPs biosensor works (Figure 2).

In previous studies, RT-LAMP-based methods have already been used to identify HIV-1 strain. Curtis et al. (2012) combined RT-LAMP with agarose gel electrophoresis for HIV-1 detection (Curtis et al., 2012). Hosaka et al. (2009) integrated with RT-LAMP with turbidimeter for HIV-1 identification, Ocwieja et al. (2015) and Zhang et al. (2022) combined RT-LAMP with fluorescence detector for HIV-1 identification. However, these techniques require special equipment for HIV-1-RT-LAMP results interpretation. Khan et al. (2023) integrated RT-LAMP with visual reagent for detection of HIV-1 through the naked eye. Nevertheless, the results were ambiguous when the RT-LAMP product concentrations were low. Their advantages/disadvantages were shown in Table 4. In our study, we first ingenious integrated RT-LAMP amplification with the AuNPs-LFA platform for the identification of HIV-1. Our AuNPs-LFA is easy to operate and cost-saving (∼US$2.0 per test). Therefore, the total cost of each HIV-1-RT-LAMP-AuNPs-LFA detection, including genomic RNA isolation (∼US$0.5), RT-LAMP reactions (∼US$1.5), and AuNPs-LFA readout (∼US$2.0), was approximately US$4.0.

**TABLE 4:** Comparisons of the commonly used RT-LAMP-based methods for detection of HIV-1.

Results detection technique	Advantages	Disadvantages	References
Agarose gel electrophoresis	High specificity and sensitivity	Require special equipment for agarose gel electrophoresis; time-consuming; need open the reaction tube for results identification	Curtis et al., 2012 Current study
Turbidity	High specificity; without opening the reaction tubes for results identification	Require special equipment for turbidity measurement	Hosaka et al., 2009 Current study
Fluorescence probe	High specificity and sensitivity; without opening the reaction tubes for results identification	Require expensive equipment for fluorescence signal detection	Ocwieja et al., 2015; Zhang et al., 2022
Visual reagent	High specificity; without opening the reaction tubes for results identification	The results are ambiguous when the LAMP product concentrations are low	Khan et al., 2023 Current study
AuNPs-LFA	High sensitivity and specificity; accurate and visual interpretation; easy to operate and cost-saving	Need open the reaction tube for results identification	Current study

Our HIV-1-RT-LAMP-AuNPs-LFA assay also has some shortcomings. First, because HIV-1 has very high heterogeneity and many genotypes, our degenerate LAMP primers specifically identify only HIV-1 genotypes (CRF01_AE, CRF07_BC, CRF08_BC, and subtype B) prevalent in China, and there is still a need to further refine the LAMP primers for detecting many more HIV-1 genotypes. Second, the outcomes obtained from the AuNPs-LFA platform with the naked eye are qualitative but not quantitative. Quantitative measurements with the HIV-1-RT-LAMP-AuNPs-LFA assay require further study. Finally, the HIV-1-RT-LAMP amplification tube must be opened to be read by the AuNPs-LFA, which will increase the risk of carry-over contamination. In our laboratory, spraying nucleic acid contamination scavenger soon after completing each AuNPs-LFA assay is an effective way to avoid nucleic acid contamination. For adaptation to clinical application, it is necessary to refine the HIV-1-RT-LAMP-AuNPs-LFA assay system and design a device that avoids the tube opening procedure to prevent aerosol contamination.

## Conclusion

Here, we integrated RT-LAMP isothermal amplification with the AuNPs-LFA platform to create a novel HIV-1-RT-LAMP-AuNPs-LFA assay system for high-sensitivity, high-specificity, rapid identification of HIV-1 by visual readout in clinical settings. Our assay had a 20-copy LoD and showed no cross-reactivity with other pathogens. The entire detection procedure can be accomplished within 45 min and with no need for any expensive facilities. Hence, our assay can meet the WHO-recommended ASSURED criteria (affordable, sensitive, specific, user-friendly, rapid and robust, equipment-free and deliverable to end users) for POC testing requirements.

## Data availability statement

The original contributions presented in the study are included in the article/Supplementary material, further inquiries can be directed to the corresponding authors.

## Author contributions

XC, WY, and JH involved in study conceptualization, supervision, and project administration. XC, CD, QiaZ, QiZ, YW, and JH performed experiments and data curation. XC and CD involved in study funding acquisition and methodology. XC, QiaZ, and YW collected clinical samples. CD, QiaZ, and QiZ involved in validation studies and visualization. XC involved in writing–original draft. JH and WY involved in writing–review and editing. All authors contributed to the article and approved the submitted version.

## Footnotes

1
http://primerexplorer.jp/e/

2
http://www.hiv.lanl.gov/content/index

3
http://www.medcalc.org/calc/diagnostic_test.php
