# Supplementary material for: Rapid and visual identification of HIV-1 using reverse transcription loop-mediated isothermal amplification integrated with a gold nanoparticle-based lateral flow assay platform
Source: Front Microbiol. 2023 Jul 12;14:1230533. doi: 10.3389/fmicb.2023.1230533 (PMC10368893; doi:10.3389/fmicb.2023.1230533)
Supplement: Supplementary file 1 [file Data_Sheet_1.pdf]

Supplementary Materials

Rapid and visual identification of HIV-1 using reverse transcription loop-mediated isothermal amplification integrated with a gold nanoparticle-based lateral flow assay platform

Xu Chen[1, 2△*], Cheng Du[△3], Qiang Zhao[4], Qi Zhao[5], Yonghu Wan[6], Jun He[4] and Wei Yuan[7*]

[1]The Second Clinical College, Guizhou University of Traditional Chinese Medicine, Guiyang, Guizhou, 550003, People's Republic of China

[2]Clinical Medical Laboratory of the Second Affiliated Hospital, Guizhou University of Traditional Chinese Medicine, Guiyang, Guizhou, 550003, People's Republic of China

[3]Department of anesthesiology, the Second Affiliated Hospital, Guizhou University of Traditional Chinese Medicine, Guiyang, Guizhou, 550003, People's Republic of China

[4]Clinical laboratory, Guizhou Provincial Center for Clinical Laboratory, Guiyang, Guizhou, 550002, People's Republic of China

[5]Gastroenterology of the Second Affiliated Hospital, Guizhou University of Traditional Chinese Medicine, Guiyang, Guizhou, 550003, People's Republic of China

[6]experiment center, Guizhou Provincial Centre for Disease Control and Prevention, Guiyang, Guizhou, 550004, People's Republic of China

[7]Department of Quality Control, Guizhou Provincial Center for Clinical Laboratory, Guiyang, Guizhou, 550002, People's Republic of China

[△]Drs. Xu Chen and Cheng Du have contributed equally to this work.

[*]Corresponding author:

Wei Yuan, E-mail: yuanwei181@126.com

Jun He, E-mail: gzgyhjjj@163.com

Xu Chen, E-mail: xuchen1220@126.com (Handing the correspondence)

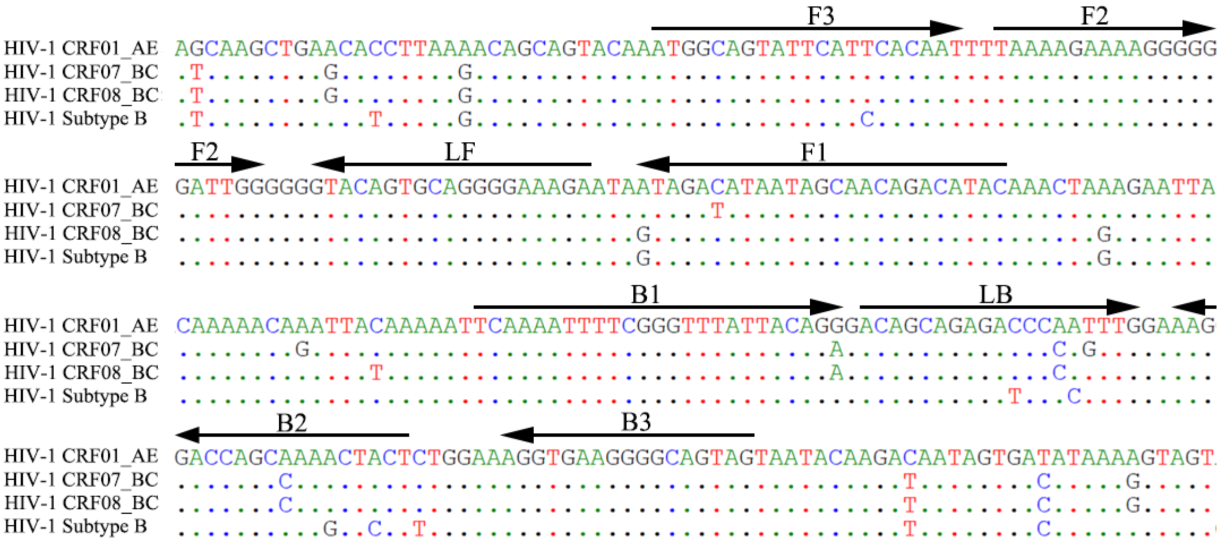

Figure S1 The nucleotide sequences and locations of the representative HIV-1 genotypes, including CRF01_AE, CRF07_BC, CRF08_BC, and subtype B *pol* genes, used to design the HIV-1-RT-LAMP primers. Right arrows and left arrows indicated the sense and complementary sequences which were used in this study, respectively.

Table S1 Comparison of RT-qPCR and HIV-1-RT-LAMP-AuNPs-LFA assays for detection of HIV-1 using clinical samples

**Sample NO.**	**RT-qPCR result （copies）**	**HIV-1-RT-LAMP-AuNPs-LFA**	**Genotype**
Test 1	2.07×10^{6}	+	CRF01_AE
Test 2	1.06×10^{2}	+	CRF01_AE
Test 3	7.67×10^{4}	+	CRF01_AE
Test 4	1.24×10^{4}	+	CRF07_BC
Test 5	3.60×10^{4}	+	CRF01_AE
Test 6	1.30×10^{5}	+	CRF01_AE
Test 7	1.65×10^{5}	+	CRF01_AE
Test 8	4.13×10^{2}	+	CRF01_AE
Test 9	6.49×10^{4}	+	CRF01_AE
Test 10	1.18×10^{3}	+	CRF07_BC
Test 11	1.00×10^{5}	+	CRF01_AE
Test 12	8.26×10^{1}	+	CRF07_BC
Test 13	1.06×10^{5}	+	CRF07_BC
Test 14	3.54×10^{3}	+	CRF08_BC
Test 15	1.36×10^{4}	+	CRF01_AE
Test 16	3.13×10^{3}	+	Subtype B
Test 17	2.60×10^{4}	+	CRF07_BC
Test 18	3.72×10^{4}	+	CRF07_BC
Test 19	1.77×10^{3}	+	CRF01_AE
Test 20	5.43×10^{5}	+	CRF01_AE
Test 21	1.71×10^{2}	+	CRF01_AE
Test 22	3.42×10^{5}	+	CRF08_BC
Test 23	2.07×10^{2}	+	CRF07_BC
Test 24	8.85×10^{4}	+	Subtype B
Test 25	1.95×10^{5}	+	Subtype B

Test 26	4.66×10^3	+	CRF01_AE
Test 27	5.90×10^4	+	CRF01_AE
Test 28	4.60×10^3	+	CRF08_BC
Test 29	2.48×10^4	+	CRF07_BC
Test 30	1.42×10^5	+	CRF07_BC
Test 31	6.49×10^5	+	Subtype B
Test 32	3.25×10^5	+	CRF07_BC
Test 33	9.44×10^4	+	CRF01_AE
Test 34	3.66×10^5	+	CRF07_BC
Test 35	1.30×10^2	+	CRF08_BC
Test 36	6.49×10^4	+	CRF07_BC
Test 37	1.42×10^2	+	CRF07_BC
Test 38	3.40×10^5	+	CRF07_BC
Test 39-65	—	—	—

Notice: The RT-qPCR diagnosis was carried out using commercial real-time TaqMan PCR Kit (DaAn Gene Co., Ltd. China). The concentrations of HIV-1 more than 30 copies will be considered as positive results according to the manufacturer’s instructions.

+, Positive; —, Negative
